# Safety Profile of *Solanum tuberosum*-Derived Exosomes: Evidence from In Vitro Experiments and Human Skin Tests

**DOI:** 10.3390/ph18040458

**Published:** 2025-03-24

**Authors:** Yeji Lee, Radwa Wahid Mohamed, Sanghwa Yang

**Affiliations:** 1 Research and Development Team, Nextab, Inc., 361, World Cup Buk-ro, Mapo-gu, Seoul 03908, Republic of Korea; 2 Department of Biochemistry and Nutrition, Women’s College for Arts, Science and Education, Ain Shams University, Cairo 11566, Egypt

**Keywords:** *Solanum tuberosum*-derived exosomes (SDEs), safety profile, wound healing, photoaging, inflammation, skin barrier

## Abstract

**Background/Objectives:** Potato (*Solanum tuberosum*)-derived exosomes (SDEs) are extracellular vesicles (66 nm in diameter) with therapeutic potential. SDEs suppress matrix metallopeptidases (*MMP*s) 1, 2, and 9, tumor necrosis factor (*TNF*), and interleukin 6 (*IL6*), while exhibiting radical-scavenging activity against the free radical 2,2-diphenyl-1-picrylhydrazyl (DPPH) in vitro and mitigating hydrogen peroxide (H_2_O_2_)-induced oxidative stress in HaCaT cells. SDEs upregulate the antioxidant gene glutathione S-transferase alpha 4 (*GSTA4*), prevent UVB damage, and regenerate photodamaged HaCaT cells. This study evaluates SDEs’ safety and skin-enhancing properties to improve their beauty-related and medical applications. **Methods:** The SDEs purified via ultracentrifugation were tested for their cytotoxic effects on HaCaT cell viability in scratch wound healing assays and for skin barrier gene modulation in HaCaT keratinocytes and Detroit 551 fibroblasts. A reverse transcription–polymerase chain reaction (RT-PCR) was used to analyze the changes in skin barrier gene expression following the SDE treatment. Cosmetic prototypes containing SDEs were assessed for skin irritation, cooling effects, periorbital wrinkle reduction, elasticity, and whitening properties. **Results:** The cytotoxicity and human topical tests confirmed the safety of SDE application. The SDEs accelerated wound closure, elevated the skin barrier gene expression level, and improved the clinical parameters, including wrinkle reduction, elasticity enhancement, and whitening. No irritation or side effects were observed. **Conclusions:** This study identified natural, edible potato-derived exosomes (SDEs) as highly safe agents that significantly enhance wound healing and promote skin barrier-related gene expression. Their multifunctional anti-aging efficacy—reducing wrinkles, enhancing elasticity, and promoting whitening without irritation—positions them as promising candidates for cosmetic and dermatological innovations. These findings warrant further exploration of SDEs for therapeutic applications, including inflammatory skin disorders and drug delivery systems.

## 1. Introduction

Photoaging represents a complex process of DNA damage induced by ultraviolet B (UVB) radiation, which subsequently leads to matrix metallopeptidase 2 (*MMP2*) production [1]. This process can occur synergistically with pathways involving the production of *MMP1*, *MMP3*, and *MMP9*, which are activated by reactive oxygen species (ROS) generated by UVB exposure [2]. Together, these pathways contribute to the mechanisms underlying wrinkle formation. The skin serves as a crucial protective barrier, performing essential functions such as providing a physical, chemical, and biological defense against environmental threats, preventing water loss, and protecting against infection.

The structural integrity and function of the skin barrier depend on the action of multiple essential genes involved in hydration, cell adhesion, and the production of structural proteins and lipids. Promoting the expression of these genes is expected to improve hydration, elasticity, and overall skin health. This is especially beneficial for addressing skin conditions involving barrier dysfunction, such as dryness, eczema, and psoriasis [3,4]. These crucial skin barrier proteins include the following: Aquaporin 3 (*AQP3*), encoding a six-transmembrane channel protein that facilitates the transportation of water and glycerol, which plays a vital role in wound healing and skin barrier maintenance [5,6]; filaggrin (FLG), which acts as a filament-aggregating protein, consolidating keratin filaments into dense bundles to enhance the cellular barrier (FLG monomers undergo proteolytic degradation into amino acids, which contributes to the synthesis of natural moisturizing factors (NMFs) essential for skin hydration, pH regulation, and UV protection [7]); and transglutaminase 1 (TGM1), an enzyme with scaffolding properties that aids in creating the cornified envelope by promoting cross-linking among structural proteins, thereby enhancing epidermal strength and stability. The mutations that inactivate human *TGM1* disrupt skin barrier function and cornified envelope formation, leading to defects in the stratum corneum. These mutations are associated with the following ailments: lamellar ichthyosis, a condition characterized by the widespread scaling and thickening of skin [8]; kallikrein-related peptidase 5 (KLK5), a sequence-specific serine protease crucial for desquamation, i.e., the shedding of corneocytes from the skin surface (this process facilitates epidermal rejuvenation and skin homeostasis and plays a role in preventing skin diseases such as harlequin ichthyosis [9,10,11]); and hyaluronan synthase genes, which are responsible for producing hyaluronan (hyaluronic acid, HA), a polysaccharide with significant water-binding properties crucial for skin hydration. Exogenous HA supplementation has been shown to improve skin conditions via hydration, wrinkle reduction, and increased elasticity [12]. Additionally, the nanoparticle-mediated delivery of hyaluronan synthase type 2 (HAS2) elevated the level of endogenous high-molecular-weight hyaluronic acid and aided in the repair of bone defects in animal models [13]. In particular, *HAS3* in the epidermis is a critical regulator of hyaluronan synthesis [14].

External factors, such as UVB radiation, wounds, and infection, continuously affect the vital functions of the skin barrier, leading to potential skin damage. Compromised skin barrier function can lead to the development of dermatological conditions such as psoriasis and atopic eczema. Psoriasis is characterized by the overexpression of *TNF* alongside the reduced expression of skin barrier genes, including *FLG* and loricrin (*LOR*) [15]. Modulating *TNF* levels may support skin barrier integrity, promote wound healing, and contribute to overall skin health [16]. The evidence suggests that *TNF* antagonists can promote the re-expression of compromised skin barrier genes, highlighting their therapeutic potential. These agents alleviate psoriatic symptoms and enhance *FLG* and *LOR* expression in human keratinocytes and patients with psoriasis in vivo [17]. This effectiveness underscores the critical link between *TNF*, skin barrier integrity, and adverse skin conditions. Moreover, skin wounds caused by burns, injuries, pressure, or friction further compromise the skin barrier. Rapid wound healing is essential for maintaining skin health by preventing dehydration, hypersensitivity, infection, and chronic inflammation [18]. Developing measures that support a healthy skin barrier and accelerate wound healing can significantly reduce the social burden and enhance the quality of life of affected patients [19].

Exosomes are extracellular vesicles composed of lipid–protein complexes, typically ranging from 50 to 120 nm in diameter, and are generated by a wide variety of eukaryotic, prokaryotic, and fungal cells. Exosomes convey biomolecules—including mRNA, miRNA, proteins, peptides, DNA, and natural small molecules—to recipient cells both in vitro and in vivo, thereby influencing gene expression [20]. Additionally, exosomes have become promising drug delivery systems for therapeutic agents, including those targeting the brain [21,22,23,24,25]. Among the various sources of exosomes, plant-derived types are particularly noteworthy. Plant exosomes exhibit physical characteristics similar to those from other sources. Plant exosomes offer distinct advantages, such as evading the immune system when introduced into animals, ensuring biocompatibility and safety, especially when derived from edible plants. Furthermore, their lower production costs make plant exosomes a promising option as next-generation carriers for exogenous medicinal agents [26,27,28]. While extracellular microvesicles share some anti-inflammatory activities with exosomes, they differ primarily in size, with the mean diameter exceeding 150 nm [29]. Recent significant advances in the cosmetic sector have highlighted the integration of food and cosmetics, leveraging bioactive substances from edible plants to enhance people’s skin health and beauty [30]. In this context, exosomes derived from edible plants, known as edible exosomes, represent promising next-generation skincare and a potential medical component due to their efficacy and safety. Nanoparticles smaller than 100 nm are particularly promising in the cosmetic industry, provided that the safety standards are met. Their small size enables them to penetrate deeper into the skin layers, initiating revitalization processes [31].

In previous reports, SDEs uniquely demonstrated therapeutic potential by suppressing *MMP*s and the inflammatory cytokines *TNF* and *IL6*, promoting keratinocyte viability, and mitigating UVB-induced photodamage. These activities are specific to SDEs, as potato protein and alcohol extracts and bacterial exosomes are not as beneficial as other SDEs, or even cause harm. These SDEs also exhibited antioxidant properties by effectively scavenging DPPH free radicals in a concentration-dependent manner and protecting HaCaT cells from H_2_O_2_-induced cytotoxicity [32]. The SDEs also effectively suppressed *IFNγ* in an ex vivo model of concanavalin-activated mouse Th1 cells, which was a model of autoimmune hepatitis [33].

Building on the previous studies, we assessed the survival rates of HaCaT cells exposed to increasing concentrations of SDEs to determine the maximum safe concentration in vitro. Additionally, the effects of SDEs on wound healing and the expression levels of skin barrier-related genes were investigated. Based on their safety profile and skin health-promoting properties, a cosmetic prototype containing SDEs as the main ingredient was then clinically tested to assess its main potential for reducing deep eye wrinkles in volunteers.

## 2. Results

### 2.1. Profile of Isolated SDEs

**Figure 1:**
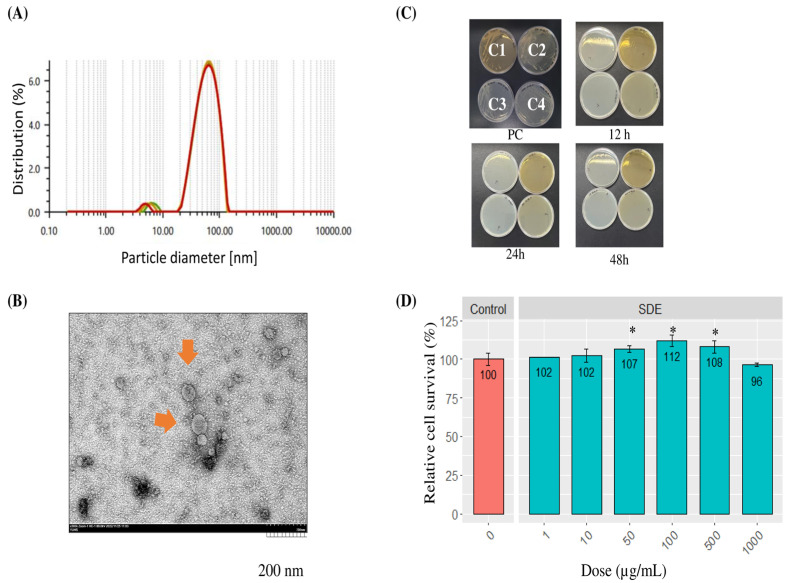
Characterization and safety assessment of SDEs. (**A**) Size distribution of SDEs analyzed by dynamic light scattering (DLS). (**B**) Transmission electron microscopy (TEM) image revealing the spherical morphology of SDEs (scale bar: 200 nm). (**C**) Bacterial contamination assessment following SDE isolation. (**D**) Cytotoxicity evaluation of SDEs on HaCaT cells treated with increasing concentrations (0–1000 µg/mL) for 24 h, as determined by cell viability using the WST-1 assay. Data are presented as mean ± SEM. * indicates statistically significant differences (*p* < 0.05) compared to ‘Control’.

The SDEs were assessed for their integrity and purity. Dynamic light scattering (DLS) analyses of three independent SDE preparations confirmed that they had a mean diameter of 66 nm with a standard deviation of 0.74 nm, which is consistent with our previous findings (Figure 1A). No contaminants or molecules larger than the exosome peak were observed, indicating a purity level exceeding 98%. Transmission electron microscopy (TEM) revealed that the SDEs exhibit double-layered, oval shapes, consistent with the general exosome morphology (Figure 1B). To prepare to evaluate the potential use of SDEs in cosmetic applications for human skin, two safety experiments were conducted. First, the SDEs were incubated on bacterial growth plates for up to 48 h, with no contamination observed. In contrast, the positive controls demonstrated the normal growth of Candida albicans on YPD plates and Staphylococcus epidermidis BRD on TSA plates. Similarly, Escherichia coli DH5α showed standard growth on both LB and MH agar plates, confirming the absence of bacterial contamination during the post-isolation manufacturing process (Figure 1C). Although bacterial contamination was already ruled out in DLS analyses, this step was performed to ensure the proper preparation of the SDE cosmetic prototype formulation for human topical application. Next, a cytotoxicity test was performed to determine the maximum safe concentration of SDEs. The HaCaT cells were treated with the SDEs at concentrations ranging from 1 to 1000 µg/mL, and cell viability was analyzed using the WST-1 assay (Figure 1D). Notably, an initial cell-growth-promoting effect was observed at a concentration of 50 µg/mL and persisted up to 500 µg/mL, reflecting cell-regenerating activity. At 1000 µg/mL, cell growth showed a slight 4% reduction relative to that of the untreated control group; however, this was not significant, suggesting that the maximum safety threshold may exceed 1000 µg/mL in vitro. Consequently, a complete median lethal dose (LD_50_) dataset could not be established. These results validate the quality control measures implemented in SDE production for human skin applications and underscore the inherent safety of SDEs.

### 2.2. Impact of SDEs on HaCaT Keratinocyte Wound Healing

**Figure 2:**
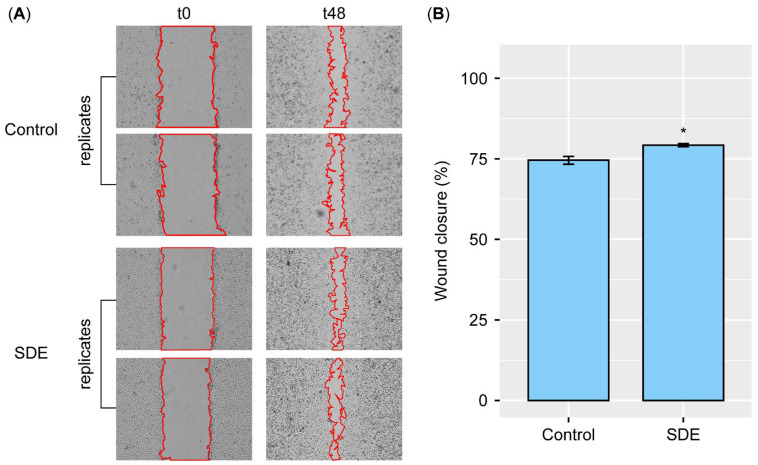
SDEs enhance wound healing in HaCaT cells. (**A**) Representative images showing closure of artificial wounds in HaCaT cells treated with either PBS (control) or SDEs over a 48 h period. (**B**) Bar graph illustrating a significantly higher percentage of wound closure observed in SDE-treated cells compared to the control group at 48 h. * indicates statistically significant differences at *p* < 0.05.

The SDEs in phosphate-buffered saline (PBS) were applied to artificial wounds on the HaCaT cells, and their wound healing activities were compared with those of the PBS sample (labeled as the ‘Control’). When the wounded area was measured over time (Figure 2A) and the percentages of wound closure were compared (Materials and Methods) at 48 h post-treatment, the SDEs significantly enhanced the rate of wound closure compared to that of the untreated control (Figure 2B). The results demonstrate that SDEs have the potential to accelerate skin repair.

### 2.3. Impacts of SDEs on the Expression of Genes Associated with the Skin Barrier

**Figure 3:**
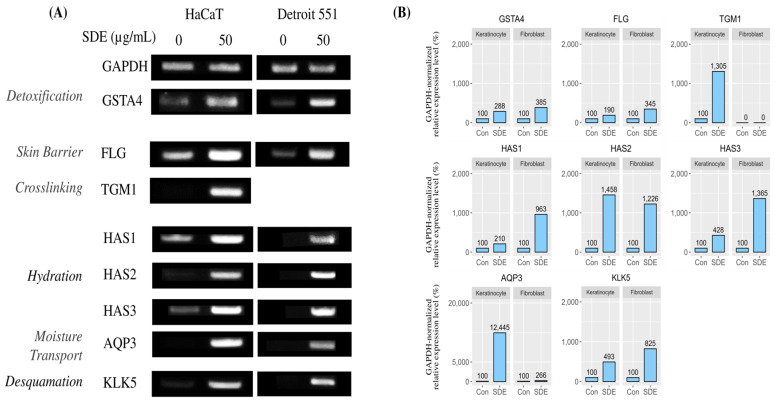
Effect of SDE treatment on skin barrier-related gene expression in HaCaT and Detroit 551 cells. (**A**) Agarose gel electrophoresis showing expression levels of *GAPDH* and various skin barrier-related genes in HaCaT and Detroit 551 cells following treatment with 50 µg/mL SDEs. (**B**) Quantification of band intensities from panel (**A**), demonstrating relative expression levels of target genes normalized to *GAPDH*. “Con” denotes the untreated control group, which received no SDE treatment and serves as a baseline for comparison.

To assess the potential effects of SDEs on skin health, we analyzed the expression of skin barrier-related genes in HaCaT keratinocytes and Detroit 551 fibroblasts following SDE treatment using RT-PCR. The resulting PCR products were separated by agarose gel electophoresis (Figure 3A), and band intensities were quantified relative to the housekeeping gene *GAPDH* to determine relative expression levels (Figure 3B). Our previous research demonstrated that SDEs (50 µg/mL) upregulate the expression of the antioxidant enzyme *GSTA4* in HaCaT cells. This effect was also replicated in Detroit 551 cells, indicating that SDEs may enhance antioxidant defenses across different cell types. Additionally, we observed that the expression level of *FLG* increased significantly, showing 3.4-fold and 1.9-fold increases in the HaCaT and Detroit 551 cells, respectively. Furthermore, *TGM1* was significantly upregulated by 13-fold in the HaCaT cells after the SDE treatment; however, RT-PCR for *TGM1* in the Detroit 551 cells was unsuccessful (Figure 3A). The SDE treatment also induced hyaluronic acid synthase (*HAS*) gene production in both the keratinocytes and fibroblasts to varying extents. *HAS2* exhibited over 10-fold upregulation in both the cell lines, while *HAS1* and *HAS3* showed greater upregulation in the Detroit 551 cells, exceeding 9- and 13-fold, respectively, compared to more modest increases of over 2- and 4-fold in the HaCaT cells following the SDE treatment. Notably, *AQP3* expression increased dramatically by at least 120-fold in the HaCaT cells after the SDE treatment, indicating a significant enhancement in potential skin hydration capabilities; conversely, *AQP3* induction in the Detroit 551 cells was more modest, with a 2.6-fold increase. Additionally, SDE administration resulted in *KLK5* upregulation by over four-fold in the keratinocytes and over eight-fold in the Detroit 551 cells. Collectively, these findings suggest that SDEs benefit skin cells by strengthening the skin barrier, increasing hydration, regulating cell turnover, and protecting against environmental damage, thereby improving skin health.

### 2.4. Clinical Skin Assessment with Prototype Cosmetics Featuring SDEs as the Primary Component

**Figure 4:**
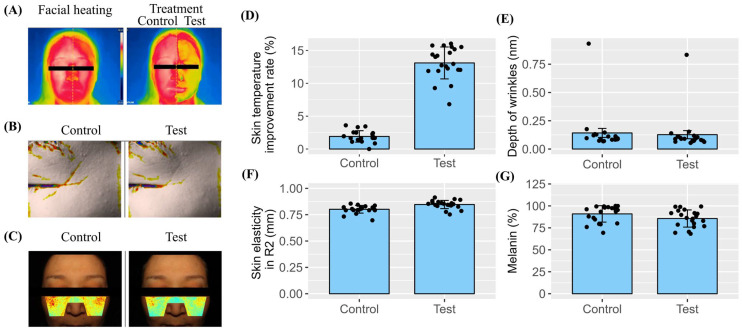
Clinical efficacy of SDE-containing cosmetic prototypes in human volunteers. (**A**) Representative images demonstrating the immediate skin cooling effect of the SDE prototype. (**B**) Representative images showing the reduction in periorbital wrinkles after 2 weeks of SDE prototype application. (**C**) Representative images illustrating the reduction in melanin levels following SDE prototype use. (**D**) Quantification of skin-cooling effects: SDE prototype vs. control (*p* < 0.001). (**E**) Comparison of periorbital wrinkle reduction between SDE prototype and control groups (*p* < 0.001). (**F**) Improvement in skin elasticity with SDE prototype treatment over 2 weeks (*p* < 0.001). (**G**) Decrease in melanin levels in SDE-treated skin compared to baseline (*p* < 0.001).

The primary finding of this skin assessment was that no adverse reactions—such as redness, edema, scaling, itching, pain, or burning sensation—were observed in any participants after initial application or throughout the subsequent two-week period (Table 1). The results demonstrated a significant cooling effect across all the patients, with a maximum improvement of 16.04%, and minimum and median rates of 6.82% and 12.96%, respectively (Figure 4A,D). A statistically significant reduction in the depth of periorbital wrinkles was reported among all the subjects, with improvements ranging from 4.76% to 25.74%, highlighting the effectiveness of the SDEs in wrinkle reduction (Figure 4B,E). Moreover, the application of prototype cosmetics containing the SDEs significantly enhanced skin elasticity (Figure 4F). Finally, a significant decrease in melanin levels was observed after two weeks of the topical administration of the SDE-containing prototypes (Figure 4G), indicating potential benefits for pigmentation issues. In summary, these findings suggest that SDEs can provide substantial benefits for skin health by enhancing cooling effects, reducing wrinkles, improving elasticity, and reducing pigmentation levels without side effects.

## 3. Discussion

This study demonstrates that the tested SDEs are safe and effective in enhancing a person’s overall skin health, including promoting wound healing, improving skin barrier integrity by upregulating skin barrier-related genes in vitro, and enhancing skin elasticity and hydration, while reducing periorbital wrinkles in human skin tests. The absence of cytotoxic effects on the HaCaT cells and irritation in the human skin applications is crucial for the development of SDEs in cosmetics, medical devices, and exosome therapeutics for future use.

Mechanistically, the SDEs enhanced HaCaT cell wound closure in the scratch assays, indicating the therapeutic potential for epithelial repair. This aligns with their ability to upregulate key skin barrier genes, including *FLG*, *TGM1*, and *AQP3*, which fortify the cornified envelope to prevent water loss and infection, while boosting hydration via water and glycerol transportation.

The dramatic 120-fold increase in *AQP3* expression highlights the unique capacity of SDEs to enhance skin moisture, which is the cornerstone of elasticity and wrinkle reduction. Additionally, the SDEs upregulated hyaluronan synthase genes (*HAS1*, *HAS2*, and *HAS3*) in both the keratinocyte and fibroblast cell lines, promoting endogenous hyaluronic acid (HA) synthesis. The elevated HA levels likely contributed to the clinical improvements in skin hydration, elasticity, and wrinkle reduction observed in the human trials. Together, these effects highlight the role of the SDEs in strengthening the epidermal barrier and maintaining skin integrity. Our previous report also demonstrated that SDEs contribute to skin health by preventing and mitigating UVB-induced photodamage through molecular mechanisms, including the downregulation of collagen-digesting *MMP*s and inflammatory cytokines (*TNF* and *IL6*), as well as antioxidant activities against DPPH and the upregulation of the antioxidant gene *GSTA4*. These clinical skin test results demonstrate the safety and efficacy of SDEs in human applications. The absence of adverse reactions, such as redness, edema, and itching, among the 21 participants confirms their safety. The significant improvement in skin elasticity, the reduction in periorbital wrinkle depth, and the decrease in melanin content after two weeks of SDE application suggest that SDEs are a valuable ingredient in anti-aging and skin-repair formulations. The cooling effect observed immediately after application suggests that SDEs provide soothing benefits, making them suitable for sensitive skin types. While our results are promising, this study has several limitations. First, this clinical trial was conducted over a relatively short period of two weeks, and the long-term effects of SDE application require further investigation. Second, this study involved a small cohort of 21 volunteers, and larger-scale trials are needed to generalize the findings.

Plant exosomes, specifically those from edible plants, are characterized by low-level immunogenicity and strong safety profiles, making them promising candidates for therapeutic innovation. Accordingly, the benefits of plant exosomes are being increasingly reported, with over 28 plant-derived exosomes—including those from edible plants, such as cabbage, grapefruit, ginger, lemon, and ginseng—exhibiting no toxicity. These exosomes have primarily been studied for their anti-inflammatory, anti-tumor, and gut microbiota-modulating properties. For example, ginger exosome-like nanoparticles (GELNs) have been reported to inhibit lung inflammation caused by exosomes from SARS-CoV-2-infected lung epithelial cells [34]. Garlic exosome-like nanoparticles (GELNs), when orally administered to mice with DSS-induced colitis, effectively alleviated colitis symptoms, including statistically significant reductions in the *TNF* and *IL6* levels in mouse sera, and shifted the composition of the intestinal microbiota to a more favorable state in the host mice [35]. However, the direct comparison of the anti-TNF and anti-IL6 activities of these plant exosomes is hindered by methodological heterogeneity across studies, including differences in experimental models (e.g., in vitro vs. in vivo) and dosing regimens.

Our approach to evaluating the safety and efficacy of exosomes began with screening their ability to suppress *TNF* expression. We compared the exosomes derived from pear (*Pyrus pyrifolia*), radish (*Raphanus sativus*), citrus (*Citrus sinensis*), date (*Phoenix dactylifera*), ginseng (*Panax ginseng*), yeast (*Saccharomyces cerevisiae*), apple (*Malus domestica*), and an intestinal microbiome source (*Lactobacillus rhamnosus*), using a consistent protocol for isolation, quantification, and cell treatment [32,33]. Our study demonstrated that potato-derived exosomes (SDEs) were unique in suppressing *TNF*, *IL6*, and MMPs while elevating the antioxidant gene *GSTA4* and skin barrier genes, providing an excellent basis for skin barrier integrity (*FLG* and *TGM1*), moisture retention (*AQP3* and *HASs*), and desquamation or dead cell shedding (*KLK5*). This makes SDEs one of the most suitable exosomes for skin cosmetic applications.

In human skin tests, the SDEs at 100 ppm demonstrated no skin irritation, including erythema, edema, squama, itching, tingling, burning, and stiffness. The in vitro cytotoxicity assays revealed that at up to a 10-fold higher concentration (1000 ppm), the SDEs did not significantly affect HaCaT cell survival, highlighting their high safety margin. Based on their safety profile and efficient cell permeability, we are now investigating whether SDEs can actively alleviate or reduce skin inflammation as a topical agent. Inflammatory skin diseases can be caused by various factors, including infection and autoimmune conditions, leading to acute and chronic skin conditions, respectively. Patients with cancer undergoing targeted therapies, cytotoxic chemotherapy, radiation therapy, or immune checkpoint inhibitors often experience skin toxicity, including xerosis (dry skin), which affects up to 84% of patients. These toxicities can cause pain, reduce their quality of life, and sometimes necessitate treatment interruptions. To address these issues, the Association Francophone des Soins Oncologiques de Support (AFSOS) and the Multinational Association of Supportive Care in Cancer (MASCC) recommend protocols to maintain hydration, skin barrier integrity, and skin microbiota diversity [36]. Given their proven safety, the ability to accelerate wound healing, the promotion of skin barrier-related gene expression, and the suitability for large-scale production, SDEs are well suited for development as dermocosmetics. Furthermore, SDEs have been shown to strongly inhibit IFNγ in an ex vivo model of autoimmune hepatitis (AIH). These combined beneficial properties make SDEs a promising candidate for development as dermocosmetics to alleviate the skin toxicities caused by various therapeutic drugs.

Future research should explore the molecular pathways through which SDEs exert their effects, particularly their interactions with the key signaling pathways involved in skin barrier function and wound healing. Exosomes from edible potatoes—namely, SDEs—present a unique opportunity for development in human medical applications, including drug delivery systems.

## 4. Materials and Methods

### 4.1. Reagents

Detroit 551 human fibroblast cells (cat. no. 10110) were purchased from Korean Cell Line Bank (Seoul, Republic of Korea). Keratinocyte HaCaT cells (cat. no. 300493) were purchased from CLS (Cell Lines Service GmbH, Eppelheim, Germany). Fetal bovine serum (FBS, heat inactivated, cat. no. 12106C), phosphate-buffered saline (PBS, pH 6.8~7.5, cat. no. LB004-01), 100× penicillin–streptomycin (10,000 units/mL, 10,000 µg/mL, respectively, cat. no. LS 202-02), Trypsin–EDTA (cat. no. LS 015-01), Minimum Essential Medium Eagle (MEM, cat. no. LM007-01), and Dulbecco’s Modified Eagle’s Medium (DMEM, cat. no. LM 001-07) were procured from WELGENE Inc. (Gyeongsangbuk-do, Republic of Korea). Superscript II (catalog number 18064022) and Pierce™ Coomassie (Bradford) Protein Assay Kit (catalog number 23200) were procured from Thermo Fisher (Waltham, MS, USA). The Cell Proliferation Reagent WST-1 (catalog number 5015944001) was procured from Sigma-Aldrich (Burlington, MA, USA). The RNeasy Mini Kit (catalog number 74004) was obtained from Qiagen GmbH (Hilden, Germany). Oligonucleotide PCR primers were procured from Bionics (Seoul, Republic of Korea).

### 4.2. Preparation and Characterization of SDEs

To purify the SDEs, potatoes were thoroughly rinsed with distilled water and then with phosphate-buffered saline (PBS, pH 7.4), followed by semi-drying. The juices were extracted using a commercial juicer in an environment that minimized heat production during processing. The extracts were successively centrifuged at 6000× *g*, 10,000× *g*, and 39,000× *g*, each for 1 h at 4 °C. The resulting supernatant was filtered using 0.8 µm cellulose nitrate membrane filters. Finally, exosomes were isolated by ultracentrifugation at 120,000× *g* for 2 h at 4 °C. The exosome pellets were resuspended in PBS (pH 7.4), and the protein concentration of the purified exosomes was determined using the Pierce™ Coomassie (Bradford) Protein Assay Kit. After the extraction of SDEs using ultracentrifugation, their size and morphology was assessed using a Litesizer DLS 500 (Anton Paar, Graz, Austria) with dynamic light scattering, along with morphology assessment via transmission electron microscopy (TEM), as detailed in previous works [32]. After ultracentrifugation, the exosome preparations were subjected to filtration using 0.2 µm sterile filters to eliminate any biological contaminants.

### 4.3. Cell Culture and SDE Administration

HaCaT and Detroit 551 cells were cultured in DMEM at a density of 5 × 10^3^ cells per well in 96-well plates, supplemented with 10% FBS, 100 U/mL penicillin, and 100 µg/mL streptomycin, at 37 °C in a 5% CO_2_ incubator. For the cell proliferation assay, SDEs were added at a final concentration of 50 µg/mL and incubated for 24 h before adding the Cell Proliferation Reagent WST-1. Formazan production was measured at 420 nm using a microplate reader.

### 4.4. Reverse Transcription–Polymerase Chain Reaction (RT-PCR)

Total RNA was extracted from HaCaT cells using the RNeasy Mini Kit. A reaction mixture of 13.4 µL, containing 1 µg of total RNA and 0.5 µg of oligo-dT primers, was incubated at 65 °C for 10 min. Superscript II (400 U), DTT, and dNTPs were added to a final volume of 20 µL and incubated at 42 °C for 90 min. The MiniAmp Plus PCR system (Thermo Fisher) was used for gene-specific amplifications, which included an initial denaturation at 95 °C for 5 min, followed by 30 cycles of denaturation at 94 °C for 30 s, annealing at 48 °C for 30 s, and extension at 72 °C for 60 s, with a final incubation at 72 °C for 10 min. The oligonucleotide primer sets used for RT-PCR are listed in Table 2. The intensity of agarose gel electrophoretic bands was analyzed using ImageJ (version 2; National Institutes of Health, Bethesda, MD, USA) and quantified by plotting the data using the ggpubr package in R 4.3.1 for Windows, with RStudio (version 2023.06.0+421; http://www.rstudio.com/) as the integrated development environment.

### 4.5. Wound Healing Assays

HaCaT cells were seeded at a density of 5 × 10^5^ cells per well in 6-well plates containing DMEM supplemented with 10% FBS, 100 U/mL penicillin, and 100 µg/mL streptomycin. The cells were incubated for 24 h at 37 °C in a 5% CO_2_ incubator. An artificial scratch was then created using a 200 µL pipette tip, and the cells were washed three times with PBS. SDEs were added at a final concentration of 50 µg/mL. The JuLI™ Stage Real-Time Cell History Recorder was used to capture live cell images at 0, 12, 24, 36, and 48 h after SDE addition. The percentages of wound closure were calculated as follows, where t_0_ represents the time of wound infliction, and t_n_ represents the time after SDE addition.

$$Wound\;Closure\;\%=\left(\frac{\left(Wound\;surface\;area\right)t_{0}-\left(Wound\;surface\;area\right)t_{n}}{\left(Wound\;surface\;area\right)t_{0}}\right)\times 100\%$$

### 4.6. Dermatological Clinical Assessment

The study was conducted in accordance with the guidelines of the Declaration of Helsinki and approved by the Institutional Review Board of Human Co., Ltd. Skin Clinical Trial Center (Seoul, Republic of Korea; protocol code HE-P23-0066, approval date: 24 March 2023). The clinical study was conducted from 3 April to 17 April 2023 at the Human Co., Ltd. Skin Clinical Trial Center (Seoul, Republic of Korea). Informed consent was obtained from all subjects involved in the study, and written informed consent was obtained from the participants to publish this paper. The study was conducted to evaluate the efficacy of SDEs for immediate skin cooling effects after a single application, as well as improvements in skin elasticity, melasma, pigmentation, and periorbital wrinkles after two weeks of use. The study was conducted in compliance with Good Clinical Practice (GCP) guidelines, the regulations of the Ministry of Food and Drug Safety (MFDS) of the Republic of Korea, and the standard operating procedures (SOPs) of the Human Co., Ltd. Skin Clinical Trial Center (Seoul, Republic of Korea). The changes in periorbital wrinkles were evaluated. The study included 21 healthy Korean male and female volunteers aged 43 to 64 years (mean ± SD: 53.3 ± 5.9) who were not taking any medication or using cosmetics containing aspirin, anti-inflammatory agents, or antihistamines. Participants with diminished skin elasticity, pronounced periorbital wrinkles, and significant melasma and pigmentation were included in the test group. The prototype cosmetics used in this trial contained SDEs at a final concentration of 100 µg/mL. The product was applied once on the test day and twice daily (morning and evening) for two weeks. The study aimed to evaluate the immediate cooling effect of a single application of the test product, as well as improvements in skin elasticity, reduction in melasma and pigmentation, and alleviation of periorbital wrinkles after two weeks of use. Assessments and measurements were conducted in a climate-controlled chamber after a 30 min rest. The room was maintained at 22 ± 2 °C and 50 ± 5% relative humidity. The skin-cooling effects were evaluated by comparing the skin surface temperatures (°C) of both cheeks before and after facial heating, as well as after a single application of the test product. Additionally, the R2 (mm) and melanin content (%) of the cheek area were measured before and after a 2-week application of the test product to evaluate improvements in skin elasticity, melasma, and pigmentation. The average depth of periorbital wrinkles was analyzed to evaluate the product’s effectiveness in reducing wrinkles in that area. The FLIR E75 Thermal Imaging Camera (FLIR Systems, Inc., Täby, Sweden) was used to evaluate the immediate cooling effect on the skin. The Cutometer^®^ Dual MPA 580 was used to measure changes in skin elasticity. DermaVision (Opto Bio-med Co., Ltd., Seoul, Republic of Korea) was used to evaluate changes in melasma and pigmentation. The Antera 3D Camera for Skin Analysis (Miravex Ltd., Dublin, Ireland) was used to evaluate changes in periorbital wrinkles. This device analyzes the skin by capturing images of reflections at seven distinct light wavelengths using a multi-spectral LED, unlike conventional imaging technologies that rely on three colors (RGB). A 3D image is generated using optical techniques and advanced mathematical algorithms, enabling accurate analysis of the skin’s texture, fine lines, wrinkles, melanin levels, and hemoglobin levels.

## 5. Conclusions

This study provides strong evidence for the safety and efficacy of *Solanum tuberosum*-derived exosomes (SDEs) in promoting skin health. To ensure the safety of SDEs for human skin applications, several precautions were taken. The organic-certified potatoes were purchased as a raw material to eliminate the risk of pesticide contamination. Additionally, NH Nonghyup Hanaro Mart, a national specialty chain store for agricultural and aquatic products from which the potatoes were sourced, conducts tests for freshness and radioactivity. In the in vitro cytotoxicity test, the SDEs did not affect skin cell growth, even at 1000 ppm (1000 µg/mL), confirming their non-cytotoxicity. This concentration is 20 and 10 times higher than the doses used in in vitro efficacy tests (50 ppm) and the SDE prototype cosmetics used for human skin tests (100 ppm), respectively.

Our in vitro analyses demonstrated that the SDEs significantly enhance wound healing in keratinocytes, suggesting their potential to support accelerated and more effective skin repair, while preventing complications such as infection and maintaining overall skin health. Mechanistically, the SDEs strongly upregulated the key skin barrier-related genes in keratinocyte and fibroblast cell lines, including *FLG*, *TGM1*, *AQP3*, *HAS1*, *HAS2*, *HAS3*, and *KLK5*. This indicates their ability to enhance skin health by strengthening the skin barrier (*FLG* and *TGM1*) to protect against environmental damage, improving skin hydration (*FLG*, *AQP3*, *HAS1*, *HAS2*, *HAS3*, and *TGM1*), and promoting balanced exfoliation, thus preventing excessive buildup (*KLK5*). These processes are crucial for healthy skin turnover, which can be impeded by aging, skin conditions, and external factors, such as UVB radiation, wounds, and infection.

Beyond wound healing, the SDEs also exhibited previously reported anti-inflammatory properties, as evidenced by the strong downregulation of inflammatory cytokines *TNF*, *IL6*, and *IFNγ* in the in vitro and ex vivo assays. Therefore, SDEs are expected to contribute to overall skin health by enhancing hydration, elasticity, and barrier function, making them particularly beneficial for conditions involving barrier dysfunction, such as dryness, eczema, and psoriasis.

The results from the human skin applications of topical prototypes containing SDEs as a key ingredient were consistent with the in vitro findings. The participants experienced significant benefits, including an immediate cooling effect, a marked reduction in periorbital wrinkles, improved skin elasticity, and decreased melanin levels. Most importantly, no adverse reactions or skin irritation were observed during this study, underscoring the safety of SDEs for topical use. The unique multifunctional properties of SDEs—spanning wound healing, barrier reinforcement, hydration, wrinkle reduction, and skin tone improvement—position them as a highly versatile ingredient for next-generation esthetic and dermatological formulations. Furthermore, their favorable safety profile and biocompatibility make them a promising candidate for innovative drug delivery systems, offering a potential alternative to animal-derived or synthetic options. Despite the safety measures and efficacy results presented in this study, additional strategies must be implemented before SDEs can be applied in injectable medical devices, therapeutics, or drug delivery systems. Therefore, future work will focus on comparing the safety, efficacy, and yield of SDEs across different strains (varieties) of *Solanum tuberosum* to identify the optimal strain. Establishing an independent or collaborative cultivation system to maintain a stable supply of SDEs from the selected strain will be crucial in addressing the regulatory hurdles before conducting human medical trials.

## Figures and Tables

**Table 1:** Skin adverse reaction statistics (*n* = 21).

Skin Adverse Reaction	After One-Time Use	After 2 Weeks
1. Erythema	0	0
2. Edema	0	0
3. Squama	0	0
4. Itching	0	0
5. Tingling	0	0
6. Burning	0	0
7. Stiffness	0	0

**Table 2:** Forward and reverse primer sequences for RT-PCR. Size (bp) refers to the size of the PCR products in base pairs.

Symbol	Name	Size (bp)	Primer (5′ → 3′)
*GAPDH*	Glyceraldehyde 3-phosphate dehydrogenase	380	ATTCCATGGCACCGTCAAGG
			TGATGGCATGGACTGTGGTC
*GSTA4*	Glutathione S-transferase alpha 4	332	GAGGGGACACTGGATCTGCT
			GGAGGCTTCTTCTTGCTGCC
*FLG*	Filaggrin	458	AGTGAGGCATACCCAGAGGA
			CCAAACGCACTTGCTTTACA
*TGM1*	Transglutaminase 1	413	AATCCTCTGATCGCATCACC
			GTGGTCAAACTGGCCGTAGT
*HAS1*	Hyaluronan synthase 1	459	ACTCGGACACAAGGTTGGAC
			TGTACAGCCACTCACGGAAG
*HAS2*	Hyaluronan synthase 2	463	TTTGGGTGTGTTCAGTGCAT
			TAAGGCAGCTGGCAAAAGAT
*HAS3*	Hyaluronan synthase 3	493	AGAAACCCGTGACCACTGAC
			ACCATCGAGATGCTTCGAGT
*AQP3*	Aquaporin 3	427	CACACGATAAGGGAGGCTGT
			CCCTCATCCTGGTGATGTTT
*KLK5*	Kallikrein related peptidase 5	554	TGTGACCACCCCTCTAACAC
			TCCTCGCACCTTTTCTGACT

## Data Availability

The data analyzed in this study are available from the first or corresponding author upon reasonable request.
